# Bivalirudin-based versus conventional heparin anticoagulation for postcardiotomy extracorporeal membrane oxygenation

**DOI:** 10.1186/cc10556

**Published:** 2011-11-20

**Authors:** Marco Ranucci, Andrea Ballotta, Hassan Kandil, Giuseppe Isgrò, Concetta Carlucci, Ekaterina Baryshnikova, Valeria Pistuddi

**Affiliations:** 1 Department of Cardiothoracic and Vascular Anesthesia and Intensive Care, IRCCS Policlinico San Donato, Via Morandi 30, 20097 San Donato Milanese (Milan), Italy

## Abstract

**Introduction:**

Extracorporeal membrane oxygenation (ECMO) after cardiac operations (postcardiotomy) is commonly used for the treatment of acute heart failure refractory to drug treatment. Bleeding and thromboembolic events are the most common complications of postcardiotomy ECMO. The present study is a retrospective comparison of the conventional heparin-based anticoagulation protocol with a bivalirudin-based, heparin-free protocol. Endpoints of this study are blood loss, allogeneic blood product use, and costs during the ECMO procedure.

**Methods:**

A retrospective study was undertaken in the setting of cardiac surgery, anesthesia, and intensive care departments of a university research hospital. Twenty-one patients (12 adults and nine children) who underwent postcardiotomy ECMO from 2008 through 2011 were retrospectively analyzed. The first consecutive eight patients were treated with heparin-based anticoagulation (H-group) and the next 13 consecutive patients with bivalirudin-based anticoagulation (B-group). The following parameters were analyzed: standard coagulation profile, thromboelastographic parameters, blood loss, allogeneic blood products use, thromboembolic complications, and costs during the ECMO treatment.

**Results:**

Patients in the B-group had significantly longer activated clotting times, activated partial thromboplastin times, and reaction times at thromboelastography. The platelet count and antithrombin activity were not significantly different, but in the H-group a significantly higher amount of platelet concentrates, fresh frozen plasma, and purified antithrombin were administered. Blood loss was significantly lower in the B-group, and the daily cost of ECMO was significantly lower in pediatric patients treated with bivalirudin. Thromboembolic complications did not differ between groups.

**Conclusions:**

Bivalirudin as the sole anticoagulant can be safely used for postcardiotomy ECMO, with a better coagulation profile, less bleeding, and allogeneic transfusions. No safety issues were raised by this study, and costs are reduced in bivalirudin-treated patients.

## Introduction

Extracorporeal membrane oxygenation (ECMO) is widely used for the circulatory and/or respiratory support of adult and pediatric patients in a number of clinical situations, including end-stage cardiac failure, acute heart failure, acute respiratory failure, and cardiopulmonary resuscitation [1-5].

Postcardiotomy ECMO for the treatment of acute heart failure following cardiac operations presents specific challenges in anticoagulation management. When the ECMO system is implanted at the end of the heart operation, or in the first postoperative hours, the deleterious effects of cardiopulmonary bypass on the coagulation system are still active, and both massive bleeding and thromboembolic complications are common events [6,7]. In a recent series of 108 cardiac surgery patients requiring ECMO for postcardiotomy acute heart failure, bleeding and thromboembolic events were the most frequent causes of death [8].

The standard anticoagulation treatment for ECMO patients is based on a continuous infusion of unfractionated heparin to reach and maintain an activated clotting time (ACT) of 160 to 200 seconds. Heparin anticoagulation chronically consumes antithrombin (AT), activates platelets, and may result in nonimmune-mediated thrombocytopenia as well as in heparin-induced thrombocytopenia (HIT). HIT in ECMO patients is quite a common feature, with reported rates of 10 to 15% [9,10], and is of course a catastrophic complication of this procedure.

Starting in June 2009, we have been performing postcardiotomy ECMO in cardiac surgery patients (newborns, infants, children and adults) with a heparin-free anticoagulation management, based on bivalirudin as the sole anticoagulant. The shift from the conventional heparin-based protocol to the bivalirudin-based protocol was internally decided by consensus among cardiac surgeons and anesthesiologists after two cases of HIT in ECMO patients that were successfully treated by stopping heparin administration and starting a bivalirudin infusion. The present study is a retrospective comparative analysis of the conventional heparin-based anticoagulation (from January 2008 through May 2009) versus the bivalirudin-based anticoagulation (from June 2009 through April 2011) with a specific respect for blood loss, thromboembolic complications, allogeneic blood product use, and costs associated with postcardiotomy ECMO.

## Materials and methods

The present study was approved by the local ethics committee, and the need for informed consent from the patients was waived. At hospital admission, all patients gave written approval for the treatment of their data in an anonymous form for scientific purposes.

### Patients

The patient population comprises all of the postcardiotomy ECMO procedures performed at our institution from 1 January 2008 through 30 April 2011, for a total of 21 cases. Eight consecutive cases were treated with conventional heparin-based anticoagulation (H-group) and the following 13 consecutive cases with a heparinless, bivalirudin-based anticoagulation (B-group). Two cases where the conventional heparin-based anticoagulation was replaced by bivalirudin anticoagulation were not included in the patient population.

### Extracorporeal membrane oxygenation procedure

The following ECMO circuits have been used during the study period: for patients < 10 kg body weight, the circuit was assembled using a Biomedicus BP50 centrifugal pump (Medtronic, Minneapolis, MN, USA) and a Dideco Lilliput 2 ECMO oxygenator (Sorin Group, Mirandola, Italy) with phosphorylcholine biocompatible treatment; for patients weighing > 10 kg, different circuits have been used according to availability - the centrifugal pumps used were the Biomedicus BP80 (Medtronic), the Stockert Revolution (Sorin Group), or the Levitronix (Levitronix, Waltham, MA, USA) equipped with the Dideco EOS ECMO (Sorin Group) oxygenator with phosphorycholine biocompatible treatment. In the last 6 months a preassembled ECMO system (permanent life support; Maquet, Rastatt, Germany) with a Rotaflow centrifugal pump and a Quadrox PLS oxygenator with Bioline^® ^coating (Maquet, Rastatt, Germany ) was used in adult patients. Only one patient in the B-group received treatment with this system.

In all patients there was a direct heart cannulation with the venous cannula placed in the right atrium, an additional venous cannula of smaller size in the left atrium, and the arterial cannula placed in the ascending aorta. The sternum was mostly left open, covered by an adhesive plastic membrane, but in some cases it was closed with the cannulas passed through the thorax and fixed to the chest wall.

The ECMO was usually performed at full flow, under normothermic conditions guaranteed by the oxygenator heat exchanger, a heater-cooler external group, and external heat-exchanging blankets.

### Anticoagulation protocol and transfusion policy

Anticoagulation monitoring during ECMO was obtained using the ACT, the activated partial thromboplastin time (aPTT) and kaolin-activated thromboelastography (Haemoscope, Niles, IL, USA), with target values reflecting the usual clinical practice [11]. The ACT was repeated every 4 hours and the target value was 160 to 180 seconds. The aPTT was repeated every 12 hours, with a target value of 50 to 80 seconds; the thromboelastography was repeated every 8 hours, with a target value for the *r *time placed at a minimum of 12 and a maximum of 30 minutes.

The initial anticoagulation protocol was different depending on the timing of the ECMO implant. In patients under cardiopulmonary bypass with full heparin treatment, where the ECMO was implanted due to the inability to wean the patient from the cardiopulmonary bypass, the heparin was fully antagonized with protamine after the ECMO implantation. Subsequently, the patient was observed for bleeding control. No anticoagulants were used until the patient was actively bleeding (> 4 ml/kg/hour). Once the blood loss was contained and stabilized, the anticoagulation protocol was started and modulated to stick to the optimal coagulation values previously described.

In patients where the systemic heparin used for the cardiac operation was already antagonized, and the ECMO was implanted subsequently to protamine administration, a bolus dose of heparin (100 IU/kg) was used for cannulation. Once the ECMO was established, the heparin was fully antagonized with protamine and again the patient entered the observation period for severe bleeding before starting any anticoagulation treatment. In the H-group, patients received a continuous intravenous infusion of unfractionated heparin at an initial dose of 5 to 10 IU/kg/hour depending on the bleeding rate. The dose was subsequently adjusted according to the ACT, aPTT, and *r *time values. In the B-group, patients received a continuous infusion of bivalirudin at an initial dose of 0.03 to 0.05 μg/kg/hour depending on the bleeding rate. The dose was subsequently adjusted according to the ACT, aPTT, and *r *time values. In patients with a reduced creatinine clearance, the starting dose was halved.

For both groups, the dose of heparin and bivalirudin was adjusted according to three variables: ACT, *r *time, and aPTT. Given the different daily frequency of measurement for these variables, the adjustments were driven initially by the ACT, and subsequently confirmed (or readjusted) according to the *r *time and, finally, to the aPTT.

Allogeneic blood products were administered according to a specific protocol. Packed red cells were transfused for hemoglobin values < 8 to 9 g/dl. Fresh frozen plasma was used for the treatment of active bleeding especially in the first postoperative hours. Platelet concentrates were used to maintain the platelet count > 80,000 cells/μl in the patient with active bleeding and > 50,000 cells/μl in the stable, nonbleeding patient. Fibrinogen was not commercially available in Italy during the study period and was never used. AT activity was maintained at > 60% by replenishment with purified AT.

Additional options for treating life-threatening bleeding included the use of recombinant activated factor VII and, for bivalirudin-treated patients, hemofiltration.

### Data collection

Data retrieved from the patients' file and institutional database included the following.

Demographics: age (years or months); gender; weight (kg); height (cm). Operative details: cardiopulmonary bypass duration (minutes); ECMO positioning in the operating room or intensive care unit; heparin and bivalirudin dose; ECMO duration (hours).

Standard coagulation profile: ACT (seconds); aPTT (seconds); International Normalized Ratio; platelet count (cells/μl); fibrinogen (mg/dl); D-dimers (μg/l); AT activity (%). All parameters except the ACT were measured and recorded at time 0 (T0, onset of heparin or bivalirudin infusion), and subsequently every 12 hours (T12, T24, T36, T48, T60, T72, T84, T96) during the first 4 ECMO days. The ACT was measured at bedside every 4 hours. Additional platelet count assays may be performed in severely bleeding patients. Data after the fourth ECMO day were available only for 50% of the patients and were not considered for the purposes of the present study.

Thromboelastography profile: *r *time (minutes); *k *time (minutes); α angle (degrees); maximal amplitude (millimeters). All parameters were measured and recorded at the same times as previously defined.

Bleeding and transfusions: bleeding (ml) was measured as chest drain output, and was standardized for body weight (ml/kg). The measurement was settled at 12 hours (from T0 through T12), 24 hours (from T12 through T24), 36 hours (from T24 through T36), and 48 hours (from T36 through T48). Total bleeding during the first 48 hours on ECMO was measured and expressed as milliliters per kilogram per day.

Transfusions of packed red cells, fresh frozen plasma, and platelet concentrates were standardized (ml/kg), and were measured at 12, 24, 36, and 48 hours (same time intervals as above). Total transfusions for each blood product during the whole ECMO time were measured and expressed as milliliters per kilogram per day. Total purified AT supplementation during the whole ECMO time was measured and expressed as international units per kilogram per day.

Outcome measurements: thromboembolic events; general outcome (weaned; survived at discharge; dead during ECMO; dead after weaning).

Cost analysis: a cost analysis was performed and expressed in terms of daily cost (€). The items concurring to the cost evaluation were: bivalirudin versus heparin cost; allogeneic blood product cost; and purified AT cost.

### Statistical analysis

Categorical variables are expressed as number and percentage. Continuous variables were checked for normal distribution with a Kolgomorov-Smirnov test. When normality of distribution was confirmed, they were expressed as mean with standard deviation (text and tables) or standard error (figures) of the mean. Otherwise, they are expressed as median with interquartile range.

Time-related variables were tested for between-group and within-group differences with an analysis of variance for repeated measures. Categorical variables were tested using a Pearson's χ^2^; additional tests included the Student's *t *test and the Mann-Whitney U test when appropriate.

## Results

The individual details of the 21 patients constituting the study population are shown in Table 1. Ten patients (48%) were pediatric cases. Nine patients (43%) died while on ECMO; seven patients (33%) were weaned from ECMO but subsequently died; and five patients (24%) survived at discharge.

**Table 1:** Details of the study population (21 cases of postcardiotomy extracorporeal membrane oxygenation)

Case	Age	Weight (kg)	ECMO implant site	CPB duration (minutes)	ECMO duration (hours)	Thrombotic complications	Anticoagulant	Outcome
1	Adult	105	OR	498	50	None	Heparin	Dead on ECMO
2	Adult	66	OR	322	170	None	Heparin	Dead on ECMO
3	Adult	100	OR	118	40	None	Heparin	Survived
4	Infant	2.9	OR	115	124	None	Heparin	Dead on ECMO
5	Child	15	OR	74	40	None	Heparin	Survived
6	Infant	3.6	ICU	125	110	None	Heparin	Weaned and dead
7	Newborn	2.7	OR	273	90	None	Heparin	Dead on ECMO
8	Newborn	3	ICU	317	16	None	Heparin	Dead on ECMO
9	Child	45	ICU	150	234	None	Bivalirudin	Dead on ECMO
10	Adult	60	ICU	73	88	None	Bivalirudin	Weaned and dead
11	Adult	74	OR	221	135	None	Bivalirudin	Survived
12	Adult	95	OR	180	39	None	Bivalirudin	Dead on ECMO
13	Adult	76	ICU	345	262	None	Bivalirudin	Weaned and dead
14	Adult	85	OR	198	233	None	Bivalirudin	Dead on ECMO
15	Adult	65	OR	442	69	None	Bivalirudin	Survived
16	Adult	75	OR	402	190	None	Bivalirudin	Weaned and dead
17	Adult	65	OR	315	67	None	Bivalirudin	Weaned and dead
18	Infant	7	ICU	85	175	None	Bivalirudin	Weaned and dead
19	Newborn	3.3	OR	255	110	None	Bivalirudin	Survived
20	Infant	7.6	OR	266	168	Yes	Bivalirudin	Weaned and dead
21	Newborn	2.6	OR	559	87	None	Bivalirudin	Dead on ECMO

CPB, cardiopulmonary bypass; ECMO, extracorporeal membrane oxygenation; OR, operating room.

In the H-group, one patient died on ECMO due to intractable bleeding; in this patient, recombinant activated factor VII was used without success. One patient suffered severe bleeding, chronic organ hypoperfusion, and multiorgan failure; one patient had a suspicion of cerebral thromboembolism; and one patient required a change of the centrifugal pump due to thrombi formation inside the pump head (two thromboembolic events).

In the B-group, two patients died on ECMO after 10 days (the longer time in our series) due to multiorgan failure, and one patient died due to septic shock. One patient received ECMO after being operated on for an acute ascending aorta dissection; he reached the hospital without consciousness, and was operated on under emergency conditions without a neurologic assessment. On day 2 on ECMO, an electroencephalographic control revealed no cerebral activity, and the ECMO treatment was stopped. One patient required an uneventful change of the centrifugal pump after 6 days on ECMO due to thrombi formation inside the pump head (one thromboembolic event). No patient in the B-group required hemofiltration to clear excessive doses of bivalirudin.

The seven patients who died in both groups after weaning from ECMO suffered a number of complications, including ongoing circulatory failure, sepsis, and multiorgan failure.

The details of the patient population in the two groups are shown in Table 2. Patients in the B-group were significantly older and had a significantly longer ECMO duration. They demonstrated a significantly lower blood loss, with lower requirements of fresh frozen plasma, platelet concentrates, and purified AT. Packed red cell transfusions were not significantly (*P *= 0.067) reduced in the B-group.

**Table 2:** Demographics and extracorporeal membrane oxygenation details of the patient population

Parameter	H-group (*n *= 8)	B-group (*n *= 13)	*P *value
Age (years)	13.9 ± 19	36.5 ± 29	0.045
Pediatric patients	5 (62%)	4 (31%)	0.154
Weight (kg)	37 ± 45	51 ± 34	0.446
Time on cardiopulmonary bypass (minutes)	230 ± 146	269 ± 142	0.562
ECMO positioning in the operating room	6 (75%)	9 (69%)	0.772
Time on ECMO (hours)	80 ± 52	143 ± 73	0.036
Use of intra-aortic balloon pump	1 (12%)	5 (38%)	0.336
Total bleeding (ml/kg/day)°	51 ± 46	16 ± 13	0.015
Total packed red cells (ml/kg/day)	25 (51)	15 (20)	0.067
Total fresh frozen plasma (ml/kg/day)	12 (76)	5.9 (9)	0.020
Total platelets (ml/kg/day)	33 (53)	3 (7)	0.008
Total purified antithrombin (IU/kg/day)	13 (31)	7 (13)	0.048
Cost in adults (€/day)	3,313 ± 2,818	1,807 ± 886	0.165
Cost in children (€/day)	760 ± 237	312 ± 56	0.008

Data presented as mean ± standard deviation, number (%) or median (interquartile range). ECMO, extracorporeal membrane oxygenation. ° during the first 48 hours

The cost analysis was split between adults and pediatric patients. There was a trend towards a minor cost in adults, and a significantly lower cost in pediatric patients in the B-group.

**Figure 1:**
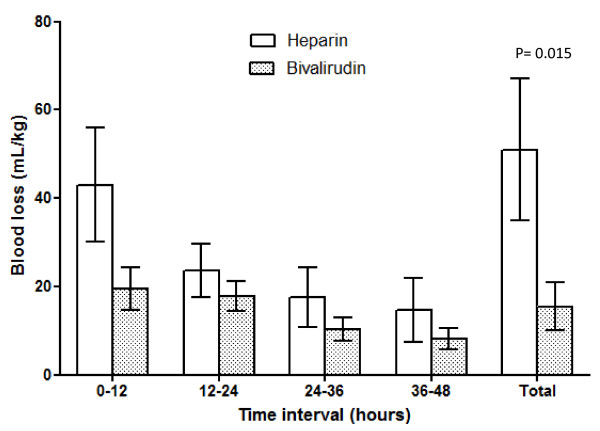
**Time-related blood loss**. Bleeding rate at 12-hour intervals (ml/kg) and total bleeding during the first 48 hours (ml/kg/day) on extracorporeal membrane oxygenation (ECMO). Data expressed as mean with standard error of the mean.

**Figure 2:**
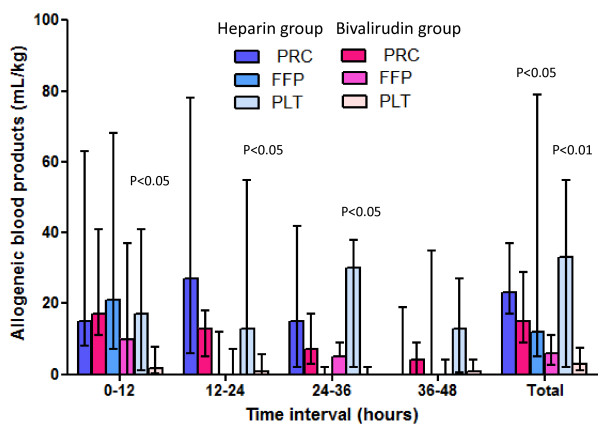
**Transfusional needs**. Allogeneic blood products used during the first 48 hours on extracorporeal membrane oxygenation (ECMO) (ml/kg) and during the whole ECMO duration (ml/kg/day). Data expressed as median with interquartile range. FFP, fresh frozen plasma; PLT, platelet concentrates. PRC, packed red cells.

Details of the time-related blood loss and transfusional needs are shown in Figures 1 and 2. Platelet concentrate transfusions were significantly lower at every time interval in the first 36 hours and during the whole ECMO period.

**Figure 3:**
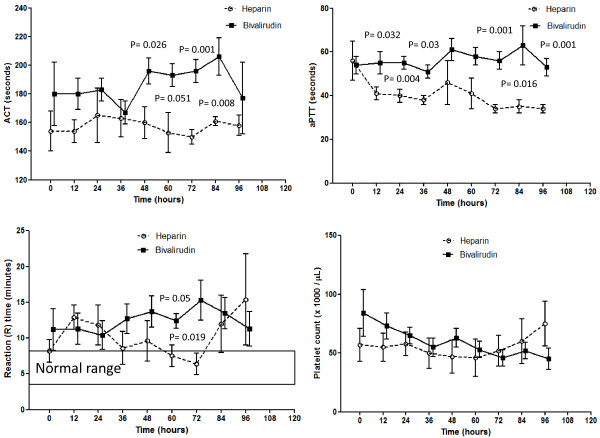
**Coagulation parameters**. Activated clotting time (ACT), activated partial thromboplastin time (aPTT), reaction (R) time at thromboelastography, and platelet count during the first 96 hours on extracorporeal membrane oxygenation. Data expressed as mean with standard error of the mean.

**Figure 4:**
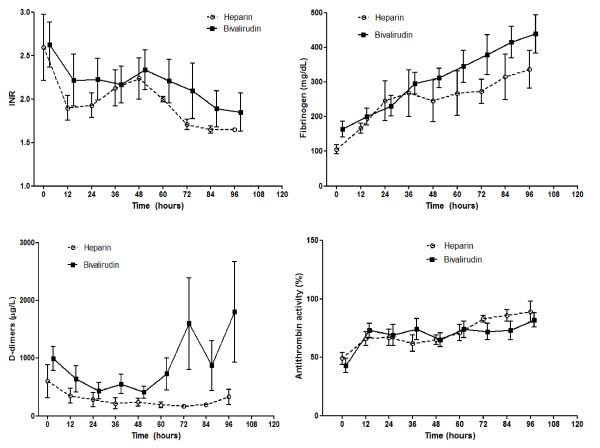
**Coagulation parameters**. International Normalized Ratio (INR), fibrinogen, D-dimers, and antithrombin activity during the first 96 hours on extracorporeal membrane oxygenation. Data expressed as mean with standard error of the mean. OK.

Coagulation parameters (Figures 3 and 4) showed significant between-group differences for the ACT (*P *= 0.015), aPTT (*P *= 0.031), and thromboelastography *r *time (*P *= 0.05) at the analysis of variance for repeated measures. The ACT was significantly longer in the B-group from T48 through T84; the aPTT was significantly longer at T12, T24, T36, T72, T84, and T96. The *r *time was significantly longer at T60 and T72 in the B-group, always being above the upper normal limit; whereas in the H-group, the value fell into the normal range at T60 and T72. The other thromboelastography-derived parameters (*k *time, α angle, and maximal amplitude ) did not differ between groups at any point in time. The platelet count did not significantly differ between groups at any point in time; notably, this was obtained using a significantly higher dose of platelet concentrates in the H-group. The nadir platelet count during the ECMO procedure was significantly (*P *= 0.022) lower in the H-group (30,600 ± 12,500 cells/μl) than in the B-group (46,900 ± 17,400 cells/μl).

The International Normalized Ratio, fibrinogen, and D-dimer levels did not differ between groups at any point in time. AT activity was similar in the two groups; however, to maintain the AT activity value, a significantly higher amount of purified AT was administered in the H-group.

**Figure 5:**
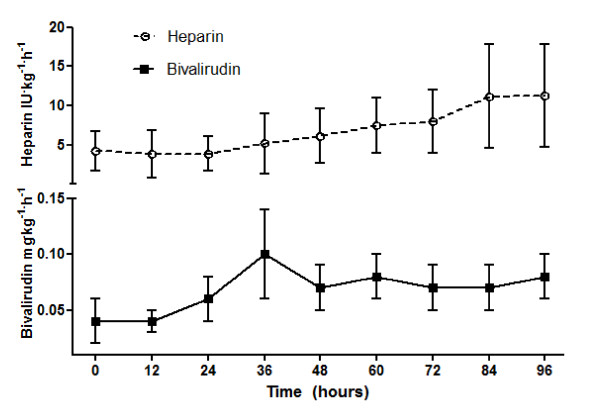
**Dose regimen of heparin and bivalirudin at different time points**. Bivalirudin and heparin dose during the first 96 hours on extracorporeal membrane oxygenation. Data expressed as mean with standard error of the mean. OK.

The dose regimen of heparin and bivalirudin at different points in time is shown in Figure 5. There was a trend towards a time-dependent dose increase for heparin, with doses ranging from 2 to 10 IU/kg/hour; the bivalirudin dose ranged between 0.05 and 0.1 mg/kg/hour.

## Discussion

Anticoagulation for postcardiotomy ECMO is still a challenging issue. In the majority of cases, ECMO is established intraoperatively or in the first postoperative hours to support failing circulation in the case of acute heart failure. Under these circumstances, the hemostatic system of the patient is affected by the effects of cardiopulmonary bypass and of the operation itself. When ECMO is implanted intraoperatively, the patient has usually been already under cardiopulmonary bypass for a long time (a median of about 4 hours in our series), due to the repeated weaning attempts. As a result, the platelet count is severely decreased, the platelet function impaired, and both coagulation factors and natural anticoagulants (AT) are consumed. Under these conditions, the patient is particularly prone to severe bleeding, and actually bleeding has been reported as the major life-threatening complication in the first hours after ECMO implantation [6,7].

Heparin has been the classical anticoagulant since the very beginning of the ECMO experiences. It is not the ideal anticoagulant for this procedure, however, for a number of reasons. Heparin is not a direct thrombin inhibitor: it requires AT to correctly antagonize thrombin, and is ineffective in blocking the clot-bound thrombin [12]. At the onset of ECMO, and throughout the whole procedure, AT values are low, and correction of low AT levels with purified AT leads to a fluctuation of the AT levels that leads to a variable pattern of heparin responsiveness, requiring several adjustments of the heparin dose [13].

Moreover, heparin is bound by the endothelial surface, bound by circulating blood cells, and avidly sequestered by platelets [13]. Again, the platelet count during the ECMO procedure is generally low and variable due to platelet concentrate administration. This pattern adds variability to the efficacy of heparin in blocking thrombin and achieving a correct anticoagulation.

Heparin itself is responsible for thrombocytopenia through nonimmune-mediated as well as immune-mediated mechanisms. HIT is quite common during ECMO procedures [9,10] and may determine an additional burden for the ECMO patient. For this reason, alternative anticoagulation strategies have been advocated by some authors [9,14].

Bivalirudin is a direct thrombin inhibitor, currently approved for anticoagulation during percutaneous coronary interventions and acute coronary syndrome. It has a short half-life of about 25 minutes and is partially cleaved by the kidney [15,16]. Bivalirudin has been safely used for cardiopulmonary bypass during cardiac operations [17] and may therefore replace heparin; however, due to its cost, the lack of an antidote, and difficult monitoring with standard ACT it is not routinely used for operative cardiopulmonary bypass.

The use of bivalirudin for ECMO procedures remains anedoctical and limited to cases of overt acute HIT during the procedure [18-20]. There is no well-established protocol for bivalirudin use in postcardiotomy ECMO patients. Koster and colleagues and Pappalardo and colleagues used a small bolus (0.5 mg/kg) followed by an infusion (0.05 to 0.15 mg/kg/hour) [19,20]. In our series, we did not use any bolus, considering the risk for severe bleeding. Even if this approach resulted in a satisfactory achievement of the target anticoagulation, further studies are required to assess whether a bolus dose is required.

To our knowledge, the present study is the only comparative study of heparin versus bivalirudin anticoagulation for ECMO patients. The main results of our study are as follows. First, patients in the B-group had a more stable anticoagulation profile, with significantly longer ACT and aPTT values maintained within the required range. Second, the platelet count and AT levels were better preserved in the B-group. Although no significant between-group differences were found for both of these variables, this was the effect of a significantly larger use of platelet concentrates and purified AT in the H-group. Third, bleeding during the ECMO period was significantly lower in the B-group, with a significantly lower need for fresh frozen plasma and platelet concentrate transfusions. Packed red cell transfusions were reduced in the B-group, but the relatively low sample size did not allow one to reach a significant between-group difference. Fourth, costs were significantly reduced in the B-group for pediatric patients. These costs were 50% reduced in adult patients, but again due to the limited sample size, without reaching a significant between-group difference. Finally, the rate of thromboembolic events was not significantly different between the two groups.

The finding that patients with a lower anticoagulation (H-group) were suffering from more bleeding and a larger need for transfusions may seem contradictory. However, inadequate thrombin suppression by heparin may generate intravascular and extravascular thrombus generation, with coagulation factors and platelet consumption, creating a condition of consumption coagulopathy. It is possible that using larger doses of heparin may result in a better control of bleeding.

Our study suggests that bivalirudin-based anticoagulation for ECMO patients is safe, effective, and limits the costs involved in an ECMO procedure. From an interpretative point of view, the starting point is that bivalirudin preserved the platelet count and AT activity better than heparin. We can hypothesize that this may be due to the absence of heparin-mediated platelet consumption and to the direct thrombin inhibition exerted by bivalirudin. The AT cofactor is not required for bivalirudin-mediated thrombin inhibition, and consequently the AT activity is better preserved. Even in the B-group, however, additional doses of purified AT were required to maintain the AT value within the normal range. This may be due to an incomplete thrombin inhibition, to a defect in hepatic AT synthesis, and to the presence of an endothelial dysfunction with release of endothelial heparinoids.

Probably as a consequence of the preservation of platelet count, bleeding was significantly contained in the B-group, therefore limiting the need for administration of allogeneic blood products. In our series, we could not diagnose any HIT event. Routine search for heparin-PF4 antibodies only recently entered into our clinical practice, however, and we cannot therefore exclude that some patients may have developed antibodies.

There are some limitations to our study. First, this is a retrospective study and therefore only generates the hypothesis that a bivalirudin-based anticoagulation for ECMO patients is superior to the classical heparin-based anticoagulation. The patient population is limited and heterogeneous for age. However, planning a randomized controlled trial in this setting of patients is hard to hypothesize. The limited amount of postcardiotomy ECMO performed in cardiac surgery institutions makes a single-center randomized trial impossible; multicenter trials are possible, but the emergency conditions that are specific for a postcardiotomy ECMO implantation make it difficult to settle a randomization process and to collect an informed consent from the relatives of the patient. However, further studies aimed to determine the correct bivalirudin dose for ECMO treatments and to confirm its safety and efficacy are warranted.

A second limitation of our study is that we are lacking data on platelet function during bivalirudin-based versus heparin-based anticoagulation, and we are also lacking important information regarding thrombin generation. Thrombin generation tests should be included in future studies to highlight the differences between the two protocols.

As a third limitation, we recognize that differences in ECMO systems may introduce a bias in the results - namely, the use of phosphorylcholine-coated (Sorin system) versus heparin-coated (Maquet system) surfaces may change the platelet count. Only one patient in the B-group received the Maquet system, however, and we therefore cannot compare subgroups based on the ECMO system used.

## Conclusions

Our study suggests that bivalirudin may be safely and effectively used in postcardiotomy ECMO to limit bleeding and allogeneic blood product transfusions. The limited size of our patient population and the limitations of our study do not allow us to claim a superiority of bivalirudin versus heparin for postcardiotomy ECMO.

Our results may be tested for other clinical situations where the ECMO is used, such as venovenous respiratory support in cases of acute lung failure recently used widely for the treatment of the H1N1 pandemic.

## Key messages

• Bivalirudin can be safely used in a heparin-free protocol for anticoagulation in postoperative ECMO patients.

• Patients treated with bivalirudin had a lower blood loss than patients treated with heparin, and required less fresh frozen plasma, platelet concentrates, and antithrombin supplementation during the ECMO period.

• In the subset of pediatric patients, significant cost containment was achieved.

• No differences in thromboembolic complications where observed in bivalirudin-treated versus heparin-treated patients.

## Abbreviations

ACT: activated clotting time; aPTT: activated partial thromboplastin time; AT: antithrombin; ECMO: extracorporeal membrane oxygenation; HIT: heparin-induced thrombocytopenia.

## Competing interests

The authors declare that they have no competing interests.

## Authors' contributions

MR was responsible for study design, statistical analysis, and manuscript preparation. AB and HK were responsible for data acquisition and interpretation. GI and CC were responsible for data acquisition and interpretation, and manuscript drafting. EB was responsible for coagulation data acquisition and manuscript preparation. VP was responsible for data acquisition and interpretation. All authors read and approved the final manuscript.
